# Training Participants to Focus on Critical Facial Features Does Not Decrease Own-Group Bias

**DOI:** 10.3389/fpsyg.2019.02081

**Published:** 2019-09-13

**Authors:** Tania Wittwer, Colin G. Tredoux, Jacques Py, Pierre-Vincent Paubel

**Affiliations:** ^1^CLLE, Université de Toulouse, CNRS, Toulouse, France; ^2^Department of Psychology, University of Cape Town, Rondebosch, South Africa

**Keywords:** training, eye-tracking, own-group bias, visual exploration, face processing

## Abstract

The own-group recognition bias (OGB) might be explained by the usage of different face processing strategies for own and other-group faces. Although featural processing appears in general to impair face recognition ability when compared to configural processing (itself perhaps a function of acquired expertise), recent research has suggested that the OGB can be reduced by directing featural processing to group-discriminating features. The present study assessed a perceptual training task intended to replicate Hills and Lewis’ (2006) findings: we trained White participants to focus more on discriminating parts of Black faces, in particular the bottom halves of the faces, expecting a reduction of the OGB as a consequence. Thirty participants completed the training task, and visual patterns of attention were recorded with an eye-tracking device. Results showed that even though participants modified their visual exploration according to task instructions, spending significantly more time on the lower halves of faces after training, the OGB unexpectedly increased rather than decreased. The difference seems to be a function of an increased false alarm rate, with participants reducing response criterion for other-group – but not own-group – faces after training.

## Introduction

The “own-group bias” (OGB) in face recognition refers to the greater facility to discriminate and recognize people of one’s own group^1^. This bias is robust and explains approximately 15% of the variance in person recognition mistakes (Meissner and Brigham, 2001). The OGB is known to be universal (Brigham et al., 2007), even though typically stronger for majority than minority groups (Wright et al., 2001).

Hancock and Rhodes (2008) have suggested that the OGB is due to differences in own and other-group face processing. Own-group faces are more likely to involve configural processing, processing the whole face and relations between features, whereas other-group faces are more likely to be processed featurally, resulting in face processing that treats features independently of each other (Michel et al., 2006a,b; Hugenberg and Corneille, 2009). Configural processing is likely a product of visual experience (Le Grand et al., 2001), and featural face processing toward other-group faces is thus likely due to a comparative deficit in perceptual experience (Diamond and Carey, 1986; Rhodes et al., 1989, 2009; Chiroro and Valentine, 1995; Walker and Tanaka, 2003; Byatt and Rhodes, 2004; Tanaka et al., 2004).

Whereas configural processing appears to occur automatically when encountering own-group faces, other-group faces appear on the contrary to invoke featural processing. Configural processing is much less present for other-group faces, while the combination of configural and featural processing could be used conjointly for own-group faces and explain the higher recognition rate of own-group faces (Hayward et al., 2008). When used on its own, featural processing does not appear to be sufficient for strong encoding, and face recognition is impaired. Even when deeper processing is involved, for instance “individuation” (Rhodes et al., 2009), it does not reduce the own-group bias. Therefore, the key may be found in a more directed and specific processing, leading people to focus on specific and critical part of faces. Indeed, the nature of feature processing could be important for successful other-group face discrimination, encoding, and recognition, if observers could use specific and expertise-related scan paths when processing faces of different groups. However, since expertise is developed mostly from exposure and experience with own-group faces, it is unlikely that observers spontaneously focus on diagnostic other-group face features (Hills and Pake, 2013) – that is, features that are efficient at individuating faces within a group.

Ellis (1975) suggested that White observers likely focus on features that individuate White people, namely eyes and hair color, whereas Black observers are likely to pay attention to individuating features of Black faces: face outline and skin color. This idea was tested (Ellis et al., 1975), and results showed that when groups of participants are asked to describe photographs of faces, they do it differently according to their group status, regardless of the stimulus group. Indeed, as predicted by Ellis (1975), White observers more often described hair (e.g., color and texture) and eye color. However, Black observers provided a more global description, more often describing hair position, eyes (size, white), eyebrows, ears, and chin. These differences, assuming that the described features are actually the ones observers pay attention to for good discrimination results, should lead to better recognition performance. An additional study (Shepherd and Deregowski, 1981) revealed that hair color, length, and texture are more likely to be described for European than African faces, while skin tone and nose breadth are more likely to be described for African than European faces, regardless of the group observers belong to (Scottish and Zimbabwean in their study). The notion of group-dependent critical features did not receive more direct support, though, until the appearance of recent studies using eye-tracking devices, and, as in the case of description of other group faces, they have revealed that differences are a function of both the observer group identity and stimulus group identity. Despite these differences, it remains that in general, eyes are commonly the features encoded first, and most frequently, regardless of stimulus group, followed by the nose and then the mouth (Althoff and Cohen, 1999; Henderson et al., 2001, 2005; Heisz and Shore, 2008; Hsiao and Cottrell, 2008). Thus, it seems that eyes appear to be key features for learning new faces (Sekiguchi, 2011). Additional but inconsistent findings in this literature are that the features focused on are dependent on the (1) observer’s group and (2) stimulus group. In the first case, White observers in several studies have focused mostly on the eyes, while Black and Asian observers have focused mostly on the nose, and then the mouth, regardless of stimulus group (Blais et al., 2008; Goldinger et al., 2009; Tan et al., 2012). In the second case, participants of all groups focused more on eyes and hair in White faces, and on noses and mouths in Black and Asian faces (Goldinger et al., 2009; Hills et al., 2013; Hills and Pake, 2013; Arizpe et al., 2016). However, according to Arizpe et al. (2016), who support the view that the features observers focus on vary by stimulus group, the differences reported in extant studies may be due to the use of different methodologies, analysis, and/or a lack of statistical power.

Bearing these considerations in mind, and especially the idea of stimulus-dependent specific critical features, some interesting work has been done on how one could use differential focus on critical features to improve face encoding and recognition of other-group faces. Thus, Howells (1938) observed higher recognition performance when only the top half of a face remained visible (as opposed to when only the bottom half remained visible), and participants were told about the importance of the eyes. Although it should be pointed out that this observed difference was not statistically tested, and that the article does not provide any information about the stimuli and participant groups. Hills and Lewis (2006) were able to eliminate an OGB in White observers with a training task that prompted focusing on critical parts of Black faces (i.e., focusing on the nose, chin, mouth and cheeks), while focusing on the non-critical features (i.e., hair style, eyes, and eyebrows) had no effect on the OGB. Subsequently, Hills and Lewis (2011), study 1, explored the effect of guided attention of White participants on critical features of Black and White faces using a fixation cross directed to either the bottom or the top half of Black and White faces. Their conclusion was that when the cross is located to the bottom part, Black faces are better encoded and recognized than White faces. However, a fixation cross located in the top face region resulted in a better performance toward White than Black faces. In the second study (Hills and Lewis, 2011; study 2), the added effect of delay between the presentation of the picture and the recognition tasks was explored with White participants. The addition of a delay resulted in a moderated or negated effect, highlighting the short-term nature of the fixation cross effect. Finally, they observed that when directing observers’ attention to the bottom half of Black faces, the OGB was reduced. Hills et al. (2013) extended these findings to Black observers. Using the same methodology with top and bottom fixation crosses, they confirmed the previous findings, namely, better performance at recognizing White faces than Black faces after a fixation cross was located in the top half of a face, while performance was better with Black than White faces after a fixation cross was located in the bottom half of a face. Crucially, they found the same patterns for Black participants, therefore resulting in the elimination of the OGB.

From these studies, we argue that to better encode a White face for later recognition, an observer should focus more on the top half of a face, and to better recognize a Black face, an observer should focus more on the bottom half of a face, regardless of the group to which the observer belongs. The present study addresses this idea directly. We aimed to decrease the OGB of White participants, presenting perceptual training during which they were asked to focus on critical discriminating features of Black faces: the bottom halves of the faces. Such a training should encourage participants to develop a deliberative way of looking at faces and involve paying greater attention to critical and diagnostic features. Participants’ awareness of critical features should be positively modified to achieve better discrimination. The increase of discriminative processing resulting from the training should transfer to encoding and thus, recognition processes. Instead of directing attention using a fixation cross (Hills and Lewis, 2011; Hills et al., 2013, study 1; Hills and Pake, 2013, study 2) the training task we constructed was an attempt to induce a spontaneous visual pattern of exploration akin to that done by Hills and Lewis (2006). We used a feature replacement technique within an eigenface software program to create differences only in the bottom halves of faces (i.e., nose, mouth, or both) in both Black and White faces. We used eye-tracking recording to establish the effect of training on the modification of visual patterns of exploration, as a manipulation check (Paterson et al., 2017). White faces were included in the present training task as stimuli, in addition to Black faces. The aim was to test if the visual patterns or exploration, while not explicitly directed by a fixation cross, would be modified in favor of an increase of the time spent on the bottom halves of Black and White faces. We expected that such an increased focus would be independent to what would spontaneously occur, which we anticipated to be preferential focus on the eyes, as found in the studies we cited earlier.

The aims of this study were thus to (1) explore the effect of attention-focused training on spontaneous visual patterns of exploration, (2) decrease the OGB through training, and (3) explore the relationship of modified visual patterns of exploration to potential decreases in the OGB. Since we used White participants, we expected to see an initial visual pattern of exploration mainly focused on the top rather than on the bottom half of the face for both own and other-group faces since that is what they would ordinarily do, when processing a face stimuli, usually White. Second, we expected participants to focus more on the bottom half than on the top half of faces as a function of the training task. Finally, we expected a reduction of the OGB after training, as a function of changed visual patterns of exploration. That is, we expected recognition for own-group faces to be unaffected by the modification of the visual patterns of exploration, but on the contrary, we expected higher recognition performance for other-group faces as a result of their greater (relative) attention to the lower halves of faces.

## Materials and Methods

### Participants

We computed required sample size with G*Power 3.1 (Faul et al., 2009). Hills and Lewis reported a very large effect size for removal of the OGB (*d* = 4.2; Hills and Lewis, 2006), which implied a sample size of 4 for our study, but an alternate calculation based on a mean difference and MSE reported on p1000 of their study resulted in an effect size of *d* = 0.8, and with alpha = 0.05, power = 0.80, and the correlation among the repeated measures conservatively at 0. With these parameters, we computed that we required 19 participants but we oversampled because we were not sure about the correlation between the measures over time, and were not entirely convinced by the effect size estimated by Hills and Lewis (2006). We thus targeted 30 participants. We recruited 39 participants (30 women, *M*_age_ = 22.77; SD_age_ = 4.99) for the study, but keeping only White participants in the analysis itself, the final sample included 30 White participants (22 women, *M*_age_ = 22.73; SD_age_ = 4.91). Participants were recruited on the campus of the University of Toulouse Jean Jaurès by direct interaction or through social media. We were interested in White participants only. However, this information was not specified during the recruitment since it is a sensitive information in France. All participants had normal or corrected-to-normal vision. The exclusion criteria of not wearing glasses or heavy make-up were made explicit during recruitment, since their presence makes eye-tracker calibration very onerous. One-third of the participants received course credit for their participation while the rest participated voluntarily, without explicit reward.

### Design

This study had a two variables factorial within-subject design: the OGB measure (before; after the training) and stimulus group (own; other-group).

### Material

#### Stimuli

A first sorting from a database of more than 500 photographs of people of a wide range of ages, collected and maintained by the second author, was made to select photographs of suitable quality (i.e., that had clearly neutral facial expressions, whose eyes were not closed, and whose frontal or three-quarter views were well standardized). From these, photographs of 140 different young males with neutral expressions (70 White and 70 Black – hereafter, respectively, referred as own and other-group) were randomly chosen: 40 for the pre-training task, 60 for the training task, and 40 for the post-training task. In both pre- and post-training tasks, 20 faces served as targets while 20 served as foils. Targets were presented from a frontal view during the encoding phase and from three-quarter view during the recognition phase, along with three-quarter view foils. The use of alternate views at encoding and recognition was intended to minimize picture recognition, and thus constitute a test of face recognition rather than picture recognition (Bruce, 1982).

For the training task, 60 trials (30 photographs of each group) were constructed. In each trial, six derivations of an original picture were generated using a face synthesis program, among which one was randomly designated as the target (ID; Tredoux et al., 2006). Synthetic faces are typically created from statistical models of real face images, and the software we used allowed feature replacement/modification holistically, through statistical sub-models of features. A trial thus consisted of the presentation of six photographs in an array: the target picture alongside the five other derived images. An image of the target identical to that in the array was presented next to the array, indicating the picture participants were asked to search for in the array. For each trial, the nose (*n* = 20), the mouth (*n* = 20), or both features (*n* = 20) were modified, to constitute the derivations.

#### Apparatus

Feature derivation photographs of the training task were generated with ID software (Tredoux et al., 2006), and controlled to be realistic and uniform so that the target did not stand out from the other array members. The training task was displayed on a 21″ Screen, with E-prime 3.0 software (Psychology Software Tools, Inc., 2016, Pittsburgh, PA). The tasks measuring the OGB were displayed on the same screen through Experiment Center 3.6 (SMI, Teltow, Germany) and eye-movements were recorded using a SMI RED250 mobile eye-tracker (SMI, Teltow, Germany) installed under the computer screen at 60 cm from the participant. The lighting in the room was identical over the sessions. The sampling rate was set at 250 Hz frequency. The calibration was effected prior to each of the OGB measurement tasks, using a 5-point calibration procedure.

### Ethics Statement

The present study received approval from the ethics committee in the University of Toulouse where the study was conducted. In addition, participants were asked to read and complete a consent form before taking part in the study.

### Procedure

**Figure 1:**
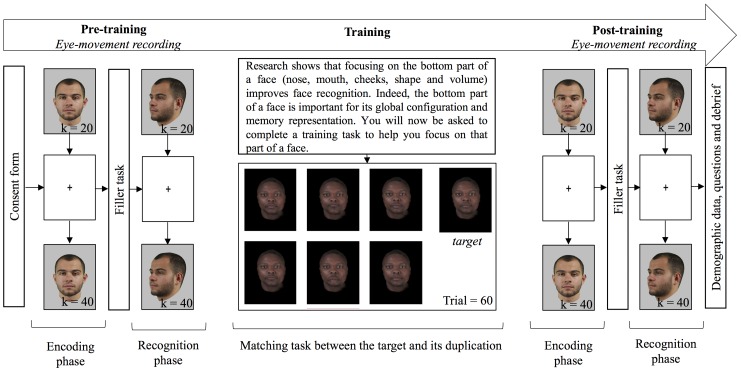
Flow chart of the procedure. Every participant completed three tasks; each task used equal numbers of faces of Black and White people. Pictures in pre- and post-training tasks are for illustration only, whereas pictures in the training tasks are examples of pictures used in the experiment.

The study was presented in three phases: two old/new recognition tasks were used as OGB pre- and post-training measures, during which eye movements were also recorded, and a training task without eye-movement recording (Figure 1). In each of the old/new recognition tasks, 20 stimuli (10 own and 10 other-group) were presented for three seconds, one second apart, in the encoding phase. A fixation cross, located in the middle of the screen was presented in-between two stimuli: approximately on the bridge of the nose for frontal stimuli and on the cheekbone for three-quarter stimuli. After a 5-min filler task (word puzzle completion), the 20 previously seen stimuli were presented again, interleaved with 20 new stimuli, in the recognition phase. Participants had to decide for each stimulus, if it had been presented or not during the first phase. No time pressure was applied. After the first old/new task, the participants completed the training task. First, to induce motivation, they were told:

Research shows that focusing on the bottom part of a face (nose, mouth, cheeks, shape, and volume) improves face recognition. Indeed, the bottom part of a face is important for its global configuration and memory representation. You will now be asked to complete a training task to help you focus on that part of a face.

Participants then completed 60 training trials: for each trial, they had to decide which of the six faces in the array matched the target. They were told that they would see several blocks of faces (i.e., trials), and that in each trial, a target face and six faces would be displayed. Their goal was to find, among the six faces, the one identical to the target. They were also told that their answer would be collected from the numeric keypad on the keyboard, and corrective feedback would be displayed. After feedback, participants were required to press the space bar to continue with the next trial. Decision time was not restricted. They were then told how to use the numeric keypad to record their answers, and started the task after an example. After each choice, a feedback popped-up as “correct” or “incorrect” with the incorrect face enclosed in a red rectangle or the correct face enclosed in a blue rectangle, where appropriate. In the case of an incorrect answer, the selected face was enclosed in a red rectangle and the right answer was displayed enclosed in a blue rectangle, and displayed simultaneously. Once all 60 trials were completed, participants completed the second old/new task with eye-movement recording, and finally answered some demographic questions. The entire experiment lasted between 45 and 90 min, and eye-tracker calibration was effected twice in the session: once before each old/new task.

### Analysis

**Figure 2:**
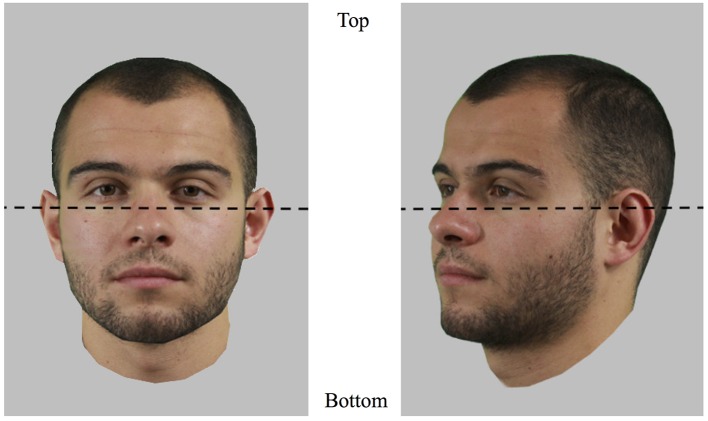
The two areas of interest (AOIs) used to assess eye movement patterns and demonstrated on frontal and three-quarter views. The coordinate of the separation is situated as close to the eyes as possible and was defined for each face individually. Pictures shown here are for illustration only.

In order to analyze the data, two areas of interest (AOIs) were defined for each face: top half versus bottom half (Figure 2). Dwell time (i.e., cumulated time of fixations) was used to express the amount of time spent on each half of the face. Analyses were performed in R (R Core Team, 2017) using the psych package (Revelle, 2017), the psycho package (Makowski, 2018), the lme4 package (Bates et al., 2015), and the lsmeans package (Lenth, 2016), conducting *post hoc* tests where appropriate. Threshold alpha for statistical significance was fixed at 5%. Cohens’ *d* values have been computed using a formula for within subject design as $d=M_{\mathrm{diff}}/\left(\left(SD_{1}+SD_{2}\right)/2\right)$ (see Lakens, 2013, formula 10).

## Results

### Visual Pattern of Exploration/Manipulation Check

Eye-movement recording failed for three participants; thus, the final sample for eye-tracking analysis was 27 participants (6 men, *M*_age_ = 22.78, SD = 5.18). A normality distribution check showed that the dwell time data were normally distributed after the training, but not before. We thus used a logarithmic transformation for both pre- and post-training data to normalize, and to allow comparisons. A mixed linear regression was run with participant as a random effect, and results showed that participants spontaneously look significantly more at the top (*M* = 3.29, SD = 0.10) than the bottom (*M* = 2.29, SD = 0.27) halves of faces before the training regardless of stimulus group, as expected (*t*(26) = 20.00, *p* < 0.001, *d* = 5.41). Then, also as expected, time spent on the bottom half of faces significantly increased from before (*M* = 2.29, SD = 0.27) to after (*M* = 2.95, SD = 0.21) training for both own and other-group faces (*β* = −0.93, *t*(182) = −13.16, *p* < 0.001, *d* = 2.75; Table 1).

**Table 1:** Mixed linear regression coefficient table, participant as random effect, and dwell time as dependent variable.

	*b*	df	Std error	*t*	*p*
Intercept	2.20	200	0.04	60.02	<0.001^***^
Top/bottom	1.09	182	0.05	21.81	<0.001^***^
Own/other	0.17	182	0.05	3.41	<0.001^***^
Pre/post	0.70	182	0.05	14.04	<0.001^***^
Top/bottom × Own/other	−0.18	182	0.07	−2.56	0.010^**^
Top/bottom × Pre/post	−0.93	182	0.07	−13.16	<0.001^***^
Own/other × Pre/post	−0.09	182	0.07	−1.28	0.202
Top/bottom × Own/other × Pre/post	0.08	182	0.10	0.76	0.446

** *p < 0.01*;

*** *p < 0.05*.

**Figure 3:**
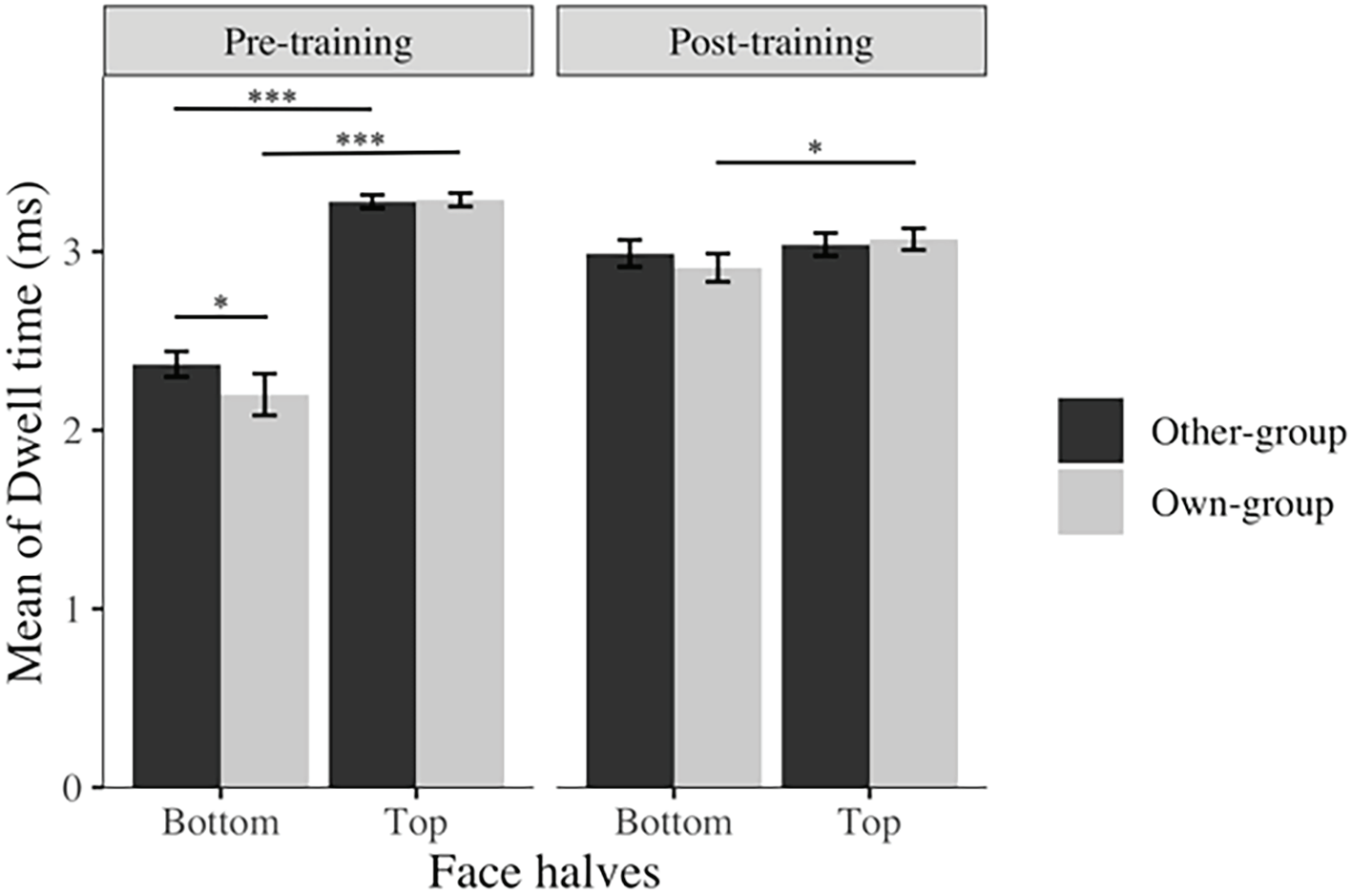
Average log transformed dwell time spent on top and bottom halves of faces across stimulus group (own; other) and time (before; after). I bars are 95% confidence intervals. ^*^p < 0.05; ^***^p < 0.001.

The training worked as expected, participants focused more on the bottom half of face after training compared to before the training, even though they still focus on the top half of faces more or equally as much as they focused on the bottom half of the face (Figure 3).

As exploratory analysis, we explored the location of the first fixation and the time to first fixation (TTFF) on the bottom halves of faces. A normality distribution check on time to first fixation revealed that it was not normally distributed, thus we used a logarithmic transformation. A mixed linear model taking proportion of first fixations located on the bottom halves of faces as dependent variable, and stimulus group and time (pre- and post-training) as fixed effects, with participants as a random effect, was run. Results showed a significant effect of stimulus group (*β* = −0.13, *t*(82) = −3.95, *p* < 0.001), of time (*β =* −0.37, *t*(82) = −11.30, *p* < 0.001), and of their interaction (*β =* 0.13, *t*(82) = 2.72, *p* = 0.008). *Post hoc* analysis revealed a significantly higher proportion of first fixations located on the bottom halves of faces after than before the training for both own-group faces (*β* = 0.24, *t*(88) = 7.25, *p* < 0.001, *d* = 2.18, see Table 2 for descriptive data) and other-group faces (*β* = 0.37, *t*(82) = 11.30, *p* < 0.001, *d* = 2.57). There were no differences between stimulus groups before the training (*β* = 0.01, *t*(82) = 0.14, *p* = 0.887, *d* = 0.29) while a higher proportion of first fixations was directed to the bottom halves of other-group rather than own-group faces after the training (*β* = 0.13, *t*(83) = 3.95, *p* < 0.001, *d* = 0.60).

**Table 2:** Mean ratio of first fixations (FFs) on bottom halves of faces to total number of first fixations (i.e., FF_bottom/FF_total), and logarithm-transformed mean time (in milliseconds) taken to first fixation (TTFF) on bottom halves of faces, by stimulus group (own; other) and time (pre; post).

	Pre-training		Post-training	
	Own-group	Other-group	Own-group	Other-group
	*M* (SD)	*M* (SD)	*M* (SD)	*M* (SD)
FF on bottom halves (mean ratio)	0.02 (0.03)	0.03 (0.04)	0.26 (0.19)	0.39 (0.24)
TTFF on bottom halves (mean)	3.11 (0.16)	3.13 (0.15)	2.69 (0.28)	2.52 (0.35)

An additional mixed linear model taking time to first fixation (i.e., time to notice a specific feature from the display of the stimulus) as dependent variable was conducted with stimulus group and time as fixed effect along with participants as random effect. Results show a very similar pattern to the previous analysis: a main effect of stimulus group (*β =* 0.16, *t*(78) = 3.37, *p* = 0.001), of time (*β =* 0.59, *t*(78) = 12.14, *p* < 0.001), and of their interaction (*β =* −0.15, *t*(78) = −2.15, *p* = 0.035). *Post hoc* analyses revealed a significantly quicker time to notice the bottom halves of faces after than before the training, for both own-group faces (*β* = −0.44, *t*(78) = −0.102, *p* < 0.001, *d* = 1.90; see Table 2 for descriptive data) and other-group faces (*β* = −0.59, *t*(78) = −12.14, *p* < 0.001, *d* = 2.44). In addition, time to notice the bottom halves of other-group faces compared to own-group faces is also significantly quicker after the training (*β* = −0.16, *t*(78) = −3.37, *p* = 0.001, *d* = 0.54) after the training, but no differences between the two groups prior to training (*β* = −0.02, *t*(78) = −0.33, *p* = 0.739, *d* = 0.13).

These results on dwell time, location of first fixations and time to first fixation, suggest that participants fixate longer on the bottom halves of faces, direct their first fixation more to the bottom halves of faces, and take less time to notice the bottom halves of faces after than before the training, indicating an effect of training on attentional eye movement patterns. The difference between own and other-group faces, revealing a higher proportion of first fixations, for longer periods of time with a quicker attention directed to the bottom halves of other-group than own-group faces suggests an effect of learning from the training: participants appear to have recognized that it is more relevant to focus on the bottom halves of other-group faces than own-group faces, and consciously adapted their strategy accordingly. In addition, participants might still use their spontaneous and more effective strategy for White faces, resulting in a similar time spent on both halves of faces.

### Own-Group Bias – Recognition Data

We computed *A*′ and *B*″, which are, respectively, non-parametric measures of discrimination and criterion bias commonly used in signal detection analyses (SDT; Stanislaw and Todorov, 1999) to assess recognition performance. Mixed linear models were tested, with participants as a random effect, to explore recognition performance across time (pre- and post-training) and across stimulus group. Results showed a significant main effect of time (*β* = −0.13, *t*(90) = −3.49, *p* < 0.001), stimulus group (*β* = 0.09, *t*(90) = 2.32, *p* < 0.001), and an interaction effect (*β* = 0.16, *t*(90) = 2.95, *p* = 0.003). On *A*′ interaction effect was also present for *B*″, namely, for the interaction between time and stimulus group (*β* = 0.19, *t*(90) = 2.15, *p* = 0.031).

**Figure 4:**
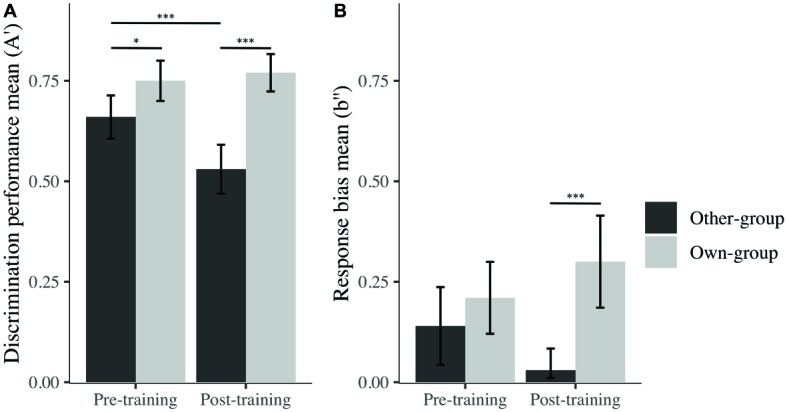
Mean of *A*′ **(A)** and *b*″ **(B)** across stimulus group (own; other) and time (pre; post). I bars are 95% confidence intervals. ^*^p < 0.05; ^***^p < 0.001.

*Post hoc* analyses were performed, and revealed the presence of an OGB before training, as expected – there was significantly higher discrimination performance (i.e., *A*′) for own than other-group stimuli (*M*_own_ = 0.75, SD = 0.14; *M*_other_ = 0.66, SD = 0.15; *β* = −0.09, *t*(93) = −2.29, *p* = 0.025, *d* = 0.62; Figure 4). However, and contrary to our expectations, the OGB became stronger after training, also showing a better performance on own than other-group stimuli (*M*_own_ = 0.77, SD = 0.13; *M*_other_ = 0.53, SD = 0.17; *β* = −0.24, *t*(93) = −6.39, *p* < 0.001, *d* = 1.60). Whereas discrimination performance for own-group stimuli was similar across time (*β* = −0.03, *t*(93) = 0.67, *p* = 0.505, *d* = 0.74), discrimination performance for other-group stimuli decreased significantly from before to after training (*β* = 0.13, *t*(93) = 3.43, *p* < 0.001, *d* = 0.81).

In terms of decision criterion, one-sample *t* tests revealed that participants presented a conservative response bias (i.e., a tendency to answer “no” more often than “yes” during the recognition task) for both stimulus groups prior to training (*M*_other_ = 0.14, SD = 0.27; *t*(29) = 2.80, *p* = 0.010, *d* = 0.51 and *M*_own_ = 0.21, SD = 0.25; *t*(29) = 4.60, *p* < 0.001, *d* = 0.85). After training, the response bias was still significantly different from zero for own-group faces (*M*_own_ = 0.30, SD = 0.32; *t*(29) = 5.10, *p* < 0.001, *d* = 0.93), while no bias was revealed for other-group faces (*M*_other_ = 0.03, SD = 0.15; *t*(29) = 1.16, *p* = 0.260, *d* = 0.21). The difference in response bias for other and own-group stimuli only became significant after training (Figure 4), congruent with the fact that there was no bias toward other-group faces, while there was a strong conservative bias toward own-group faces. These differences were nonetheless not significant on measures taken before and after the training. To summarize, participants’ discrimination performance decreased after the training for other-group faces, but at the same time, their response bias changed from conservative to unbiased toward other-group faces.

To further explore the potential reasons behind this OGB increase, separate mixed linear models were run for hits, false alarms, correct rejections, and misses (percentages) with participants as a random effect, to explore recognition performance across time (pre- and post-training) and across stimulus group. We observed an effect on correct rejections for time (*β* = −1.43, *t*(90) = −3.21, *p* < 0.001), stimulus group (*β* = 0.97, *t*(90) = 2.57, *p* = 0.010), and their interaction (*β* = 1.87, *t*(90) = 3.51, *p* < 0.001). However, no effects were found for these factors on hits. We found effects on false alarms for time (*β* = 1.43, *t*(90) = 3.88, *p* < 0.001), stimulus group (*β* = −0.93, *t*(90) = −2.52, *p* = 0.012), and their interaction (*β* = −1.93, *t*(90) = −3.70, *p* < 0.001) while observing no effect on misses for any of the factors.

**Figure 5:**
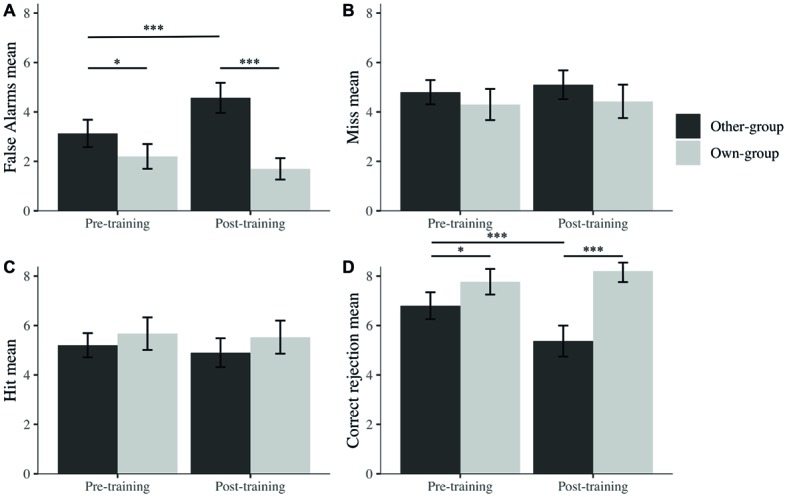
Mean percentage false alarms **(A)**, misses **(B)**, hits **(C)**, and correct rejections **(D)** across stimulus group (own; other) and time (pre; post). I bars are 95% confidence intervals. ^*^p < 0.05; ^***^p < 0.001.

It seems that changes in discrimination performance were not driven by decisions taken when the target was present (i.e., hit or miss) but by decisions taken when the picture was a foil (i.e., correct rejection or false alarms). Indeed, after *post hoc* exploration (Figure 5), we noticed that the difference in *A*′ is due to a significantly higher rate of false alarms after the training for other-group stimuli and a lower rate of correct rejection after the training for own-group stimuli. There were also significantly more false alarms toward other than own-group stimuli and more correct rejections of own than other-group stimuli. Both differences were larger after the training, in comparison to before training.

### The Modification of the Own-Group Recognition Bias as a Function of the Modification of Visual Exploration

An additional aim of this research was to explore the relationship between the OGB and the focus on the bottom halves of faces. We made the hypothesis that the OGB should decrease as a function of a modification intended by the intervention to the visual pattern of exploration. We computed the correlation between *A*′ and the time spent on the bottom half of the faces, for both own and other-group stimuli after the training. Contrary to our hypothesis, neither correlation displayed a significant relation between the time spent on the bottom half of the face and discrimination performance for both other-group (*r* = 0.36, *t*(25) = 1.92, *p* = 0.066, CI [−0.02, 0.65]) and own-group (*r* = −0.32, *t*(25) = −1.70, *p* = 0.102, CI [−0.63, 0.07]) faces, after the training. However, this could be due to a lack of power, and it is noteworthy that no significant correlations were evident prior to training for own-group (*r* = 0.03, *t*(25) = 0.14, *p* = 0.887, 95%CI [−0.36, 0.40]) or for other-group faces (*r* = − 0.06, *t*(25) = −0.32, *p* = 0.750, 95%CI [−0.43, 0.32]).

### Training Task

Even though we had no clear expectations about performance during the training task itself, it seemed useful to explore the training data as it might have shed light on the results reported above. Interestingly, participants performed significantly better on other-group than on own-group trials (*M*_other_ = 0.75, SD = 0.16; *M*_own_ = 0.36, SD = 0.11; *t*(28) = −14.10, *p* < 0.001, *d* = 2.85). Since feedback was displayed after each answer, we assume that participants were aware of their own performance.

## Discussion

In the present study, we first confirmed through eye-movement data that training participants to pay attention to the bottom halves of faces modified their visual pattern of exploration in favor of spending significantly more time focusing on the bottom halves of faces. The training program we implemented did not reverse the pattern of visual exploration, but created a better balance between time spent on the top and bottom halves of faces, with more attention being paid to the bottom halves of faces in particular, from the first fixation onward, after the training. Thus, training made participants pay more attention to the bottom halves of faces as a function of training. However, a difference appeared between processing of own-group and other-group faces. Before the training, participants focused more on the top than the bottom halves of faces, for both group of faces. After the training, they focused more on the bottom than the top halves of faces than before the training; however, the difference between dwell time on top and bottom remained significant only for own-group faces but not for other-group faces. The same pattern was found for the proportion of first fixations directed to the bottom halves of faces. These results suggest that participants learned the processing change intended by the training task, namely that focusing on the bottom halves of faces would help to increase recognition performance. These results also suggest that training raised awareness that focusing on the bottom halves of other-group faces increased their performance while it decreased it for own-group faces. Therefore, after the training and under an unconstrained visual exploration, participants directed their first fixation more to the bottom halves of other-group than own-group faces, and focused more on the bottom than top halves of faces for other-group faces while they focused equally on the two halves for own-group faces. Our results also corroborate previous findings that individuals spontaneously direct first fixations to the eyes (in our work, to the top halves of faces), even when they are asked and trained to focus on the bottom halves of faces.

However, unlike Hills and Lewis (2006), we did not observe a decrease of the OGB as a function of training. In fact, we found a significant increase of the OGB, which on closer inspection seemed due to an increase in false alarms toward other-group faces, while discrimination performance for own-group faces did not change significantly as a function of training. This increase could be explained by a training effect, albeit not one we expected. Indeed, regarding the results from the training task (i.e., better performance on other than own-group faces), we suggest that participants were aware of their higher performance for other-group faces due to the feedback they were given after each trial. In addition to this awareness, they appear to have accepted that focusing on the lower halves of faces is useful for discriminating other-group faces, highlighted by the changes in their patterns of visual exploration. However, participants may have become “over-confident” in their capacity to discriminate other-group faces, but less confident in their discrimination ability for own-group faces. This hypothesis is supported by the absence of response bias toward other-group faces after training, while a conservative bias was observed toward other group faces before training. This overconfidence in other-group face discrimination performance may have resulted in more error toward other-group faces while making more careful decisions toward own-group faces, as shown in the SDT measure of criterion, regardless of the visual strategy used for each group of faces.

The recognition of own-group faces in the present study was not affected by the time spent on the bottom halves of faces, although participants did spend more time on the bottom halves for own-group faces after training. However, one could increase the sample size and explore the promising observations made in the correlation analysis of time spent on the bottom half of faces and discrimination performance. Modifying the pattern of visual exploration does not seem to be enough to remove the OGB. Other studies may be helpful in this respect, since they often assess complementary or different strategies than mere perception, and participants can in these studies sometimes better associate faces with labels (Lebrecht et al., 2009; Tanaka and Pierce, 2009), describe faces physically (Malpass et al., 1973), rate attractiveness of faces (Hills and Lewis, 2006), or rate distinctiveness of faces (Hills and Lewis, 2011; Hills et al., 2013). Moreover, the difference between the present study and previous studies may have to do with the fact that participants were trained to develop a spontaneous way of looking at faces, and their first fixation was thus not constrained by any fixation cross (Hills et al., 2013; Hills and Pake, 2013). However, some studies have observed that the first two fixations are more important for recognition than complete gaze patterns (Hsiao and Cottrell, 2008). Finally, the present study used a different training regimen to that used by Hills and Lewis (2006), modifying only noses and/or mouths, while they modified noses, mouths, chins, and cheeks. This difference might explain the failure here to replicate their results, since their more extensive modifications could have led to a different type of processing than that which we induced.

We would like to acknowledge some limitations of the present study, and encourage further studies to address the limitations, and to pursue our findings. First, we tested White participants only, not offering a complete cross-over design. We did not include Black participants in the present study, not allowing us to explore if Black participants would have a spontaneously higher pre-training dwell time directed to the bottom halves of faces than White participants. Including Black participants could also be done in future studies, with the aim of exploring the effect of focusing on the top halves of faces, in order to eliminate an OGB from Black participants. Second, we did not use a control group, which could have added value to our results. Finally, in order to support our suggestion on the distinction we made between the effect of the instruction and the training task, one could design a study teasing out the effects of each, allowing conclusions to be drawn perhaps on the relative effect of each on the results observed in the present study.

## Conclusion

In conclusion, the present study showed that training that encouraged participants to focus on apparently diagnostic facial features of other-group faces did not assist recognition of other-group faces. In fact, such a strategy may have increased the OGB. One of the explanations could be that instead of improving face encoding by getting participants to pay attention to more discriminating features, the present training may have restricted the processing, thus reducing attention to other important parts of the face. Since own-group faces already profit from configural processing, increased time spent on the bottom halves of the face is not likely to impair discrimination performance.

In further studies, one should consider developing more thoroughgoing configural processing through training. It could also be of some interest to explore the same task but conversely: train Black participants to focus more on the top halves of faces to see whether that reduces the OGB increasing their discrimination performance for other-group faces.

## Data Availability

The datasets generated for this study are available on request to the corresponding author.

## Ethics Statement

The protocol of the present study received approval from the Comité d’Éthique de la Recherche de l’Université fédérale de Toulouse (notice n°2019-147). In addition, participants were asked to read and complete an informed consent form before taking part in the study.

## Author Contributions

TW designed, planned, and collected the data under the supervision of CT and JP. TW and P-VP conceived the different tasks. P-VP worked out the technical details and performed the extraction and manipulation of the data. TW performed the data analysis and wrote the manuscript. CT provided a review of the manuscript for language, statistical analysis, and feedback on the final version.

### Conflict of Interest Statement

The authors declare that the research was conducted in the absence of any commercial or financial relationships that could be construed as a potential conflict of interest.

## Footnotes

^1^We use the term “group” here in preference to the more commonly used term “race,” since the phenomenon occurs for groups other than “race”.
